# Genome-Wide Characterization, Expression Profile Analysis of WRKY Family Genes in *Santalum album* and Functional Identification of Their Role in Abiotic Stress

**DOI:** 10.3390/ijms20225676

**Published:** 2019-11-13

**Authors:** Haifeng Yan, Mingzhi Li, Yuping Xiong, Jianming Wu, Jaime A. Teixeira da Silva, Guohua Ma

**Affiliations:** 1 Sugarcane Research Institute, Guangxi Academy of Agricultural Sciences, Nanning 530007, China; gstsyhf@163.com; 2 Biodata Biotechnology Co., Ltd, Hefei 230031, China; Limzhi87@163.com; 3 Guangdong Provincial Key Laboratory of Applied Botany, South China Botanical Garden, the Chinese Academy of Sciences, Guangzhou 510650, China; xiongyuping@scbg.ac.cn; 4 P.O. Box 7, Miki-cho Post Office, Miki-cho, Ikenobe 3011-2, Kagawa-ken 761-0799, Japan

**Keywords:** WRKY transcription factor, sandalwood, SA (salicylic acid), MeJA (methyl jasmonate), salt tolerance

## Abstract

WRKY proteins are a large superfamily of transcription factors that are involved in diverse biological processes including development, as well as biotic and abiotic stress responses in plants. WRKY family proteins have been extensively characterized and analyzed in many plant species, including Arabidopsis, rice, and poplar. However, knowledge on WRKY transcription factors in *Santalum album* is scarce. Based on *S. album* genome and transcriptome data, 64 *SaWRKY* genes were identified in this study. A phylogenetic analysis based on the structures of WRKY protein sequences divided these genes into three major groups (I, II, III) together with WRKY protein sequences from Arabidopsis. Tissue-specific expression patterns showed that 37 *SaWRKY* genes were expressed in at least one of five tissues (leaves, roots, heartwood, sapwood, or the transition zone), while the remaining four genes weakly expressed in all of these tissues. Analysis of the expression profiles of the 42 *SaWRKY* genes after callus was initiated by salicylic acid (SA) and methyl jasmonate (MeJA) revealed that 25 and 24 *SaWRKY* genes, respectively, were significantly induced. The function of *SaWRKY1*, which was significantly up-regulated by SA and MeJA, was analyzed. *SaWRKY1* was localized in the nucleus and its overexpression improved salt tolerance in transgenic Arabidopsis. Our study provides important information to further identify the functions of *SaWRKY* genes and to understand the roles of *SaWRKY* family genes involved in the development and in SA- and MeJA-mediated stress responses.

## 1. Introduction

Transcription factors (TFs) are proteins that can bind to specific DNA target sites and activate and/or repress transcription of their target genes [1]. Among them, WRKY TFs are mainly found in plants, while a few are found in the protist *Giardia lamblia* and the slime mold *Dictyostelium discoideum* [2,3]. In green plants, the WRKY family is the eighth largest TF family following ethylene responsive factor (ERF), C_2_H_2_, basic helix-loop-helix (bHLH), myeloblastosis (MYB), NAC (NAM, ATAF1/2 and CUC2), basic leucine zipper (bZIP), and MYB-related families [4] (http://planttfdb.cbi.pku.edu.cn/). The first WRKY TF, SWEETPOTATO FACTOR1 (SPF1), was identified in sweet potato (*Ipomoea batatas*) in 1994 [5]. Since then, numerous *WRKY* genes have been identified after genome-wide analyses of plants over the past 24 years, including 74 in Arabidopsis (*Arabidopsis thaliana*) [6], 102 in rice (*Oryza sativa*) [7], 197 in soybean (*Glycine max*) [8], and 100 in poplar (*Populus trichocarpa* and *P. tomentosa*) [9]. The WRKY family is characterized by the presence of one or two conserved 60 amino acid WRKY domains (WDs), which include the highly conserved WRKYGQK sequence at its N-terminal end and a zinc-finger-like motif at the C-terminal end [6]. The C-terminal WRKY domain is mainly responsible for recognizing the cognate *cis-*acting W box elements (T)(T)TGAC(C/T) while the N-terminal end participates in increasing the affinity or specificity of their target sites [6]. Based on the number of WDs and the pattern of their zinc-finger-like motifs, all members of the WRKY family proteins have been classified into three groups I-III [6,10]. WRKY proteins with two WDs fall into group I, whereas proteins with only one WD are assigned to groups II or III. Members of groups I and II have the same type of finger motif, C-C-H-H, while members of group III contain a unique C-C-H-C motif [6]. In addition, group II proteins were further split into five distinct subgroups (IIa, IIb, IIc, IId and IIe) based on a phylogenetic analysis [6,10]. Apart from the WDs and zinc-finger-like motif, domains for transcobalamin (R) proteins also coexist with WRKY TFs forming R protein-WRKY genes in monocot genomes such as in switchgrass (*Panicum virgatum*) [11].

WRKY TFs play well-documented roles in regulating various biological processes in plants. The majority of reports demonstrated that numerous WRKY TFs are involved in transcriptional reprogramming associated with the plant’s biotic stress response. *AtWRKY3* and *AtWRKY4* expression was induced by pathogen infection and salicylic acid (SA), and the T-DNA mutants and transgenic lines of *AtWRKY3* and *AtWRKY4* suggested that they all had enhanced plant tolerance to necrotrophic pathogens. At the same time, *AtWRKY4* negatively regulated plant tolerance to biotrophic pathogens [12]. *AtWRKY38* and *AtWRKY62*, which were induced by pathogen infection and SA treatment, act as negative regulators of plant basal defense [13]. In rice, *OsWRKY03* (an *AtWRKY29/22* homolog) was involved in SA-dependent or jasmonic acid (JA)-dependent defense signaling cascades and its overexpression upregulated *OsNPR1*, *OsPR1b*, phenylalanine ammonia-lyase *ZB8* and peroxidase *POX22*.*3* [14]. Furthermore, many reports have shown that WRKY TFs are involved in abiotic stresses, including cold, wounding, heat, ultraviolet light, drought, and salinity [15,16]. In addition, WRKY TFs also play important roles in regulating plant developmental processes, such as seed dormancy and germination [17,18], root hair growth [19], and leaf senescence [20].

*Santalum album* L., commonly known as sandalwood, is a hemiparasitic tropical tree distributed in India, Indonesia, Malaysia, and Australia [21]. It is valued for its essential oil, which is extracted from aromatic heartwood and roots, and is used in aromatherapy, perfumes, cosmetics, medicine and sacred unguents [22,23]. (*Z*)-α-Santalol, (*Z*)-β-santalol, (*Z*)-*epi*-β-santalol, (*Z*)-α-*exo*-bergamotol, and sesquiterpene alcohols are the major components (70%) of *S. album* essential oil [23]. Unsustainable demand and exploitation of this slow-growing tree has threatened wild sandalwood populations in their native locations [24]. In an effort to meet the demand for its essential oil, *S. album* has been planted in Malaysia, Indonesia, Philippines, Northern Australia, and South China [25]. *S. album* was introduced to South China Botanical Garden in 1970, and is now cultivated in several subtropical regions of China such as Guangdong, Guangxi, Hainan, and Fujian provinces. Various stresses, including low temperature [26] or highly saline or alkaline soil, negatively impact the cultivation and growth of this species. The molecular biology of *S. album* has made considerable strides in recent years [27], including abiotic stress tolerance. WRKY TFs play important roles in plants against abiotic stress, so characterization and functional analysis of the WRKY family genes in *S. album* would shed light on the stress mechanisms underlying the involvement of WRKY genes, and it could also serve to breed *S. album* varieties that are resistant to these stresses. As far as we are aware, there are few reports available on WRKY TFs in *S. album*. Large-scale available RNA-seq data sets [26,28,29] and genomic data generated recently in *S. album* [30] might offer additional insight about WRKY genes.

In this study, a total of 64 *SaWRKY* genes from *S. album* were identified, and their conserved motif and tissue expression patterns were analyzed. In order to understand their evolutionary relationship, a phylogenetic tree that combined WRKY proteins from *A. thaliana* was constructed. In addition, the expression profiles of 42 *SaWRKY* genes under SA and methyl jasmonate (MeJA) treatments were generated using RT-qPCR, while the functional analysis of *SaWRKY1* (induced both by SA and MeJA) was carried out by heterologous expression in transgenic *A. thaliana*. The findings of our study will help to understand the roles of *SaWRKY* genes in SA- and MeJA-mediated pathways and to further identify the functions of this essential gene family in *S. album*.

## 2. Results

### 2.1. Identification and Subcellular Localization of SaWRKYs

A total of 64 *SaWRKY* genes named *SaWRKY1* to *SaWRKY64* were identified (Table 1) by searching the *S. album* genome and transcriptome datasets using total *A. thaliana AtWRKY* genes as queries. All of the identified SaWRKY proteins contained at least one highly conserved heptapeptide WRKYGQK domain, while 13 out of 64 SaWRKY proteins contained two WRKYGQK domains. The length of the amino acid sequence in the 64 SaWRKY proteins (Table 1, Table S1) ranged from 121 (SaWRKY45) to 764 (SaWRKY12) amino acids, with an average of 384 amino acids. A C-C-H-H type zinc-finger motif was found in 56 SaWRKY proteins whereas SaWRKY7, 9, 28, 47 and 57 had a C-C-H-C type zinc-finger motif, and other variants of zinc-finger motifs such as C-C-H-T (SaWRKY12, 53), C-C-H-L (SaWRKY25, 51), C-C-H-V (SaWRKY42), C-C-H-Y (SaWRKY45), and C-C-H-S (SaWRKY58), were also found (Table 1).

Based on a prediction by the PSORT program, the subcellular localization of most of the 64 SaWRKY proteins was in the nucleus, except for SaWRKY8, which was found in the Golgi apparatus, SaWRKY45 in the cytoplasm, SaWRKY46 in the peroxisome, and SaWRKY49 in the chloroplast (Table 1).

### 2.2. Phylogenetic Analysis of SaWRKY Proteins 

**Figure 1:**
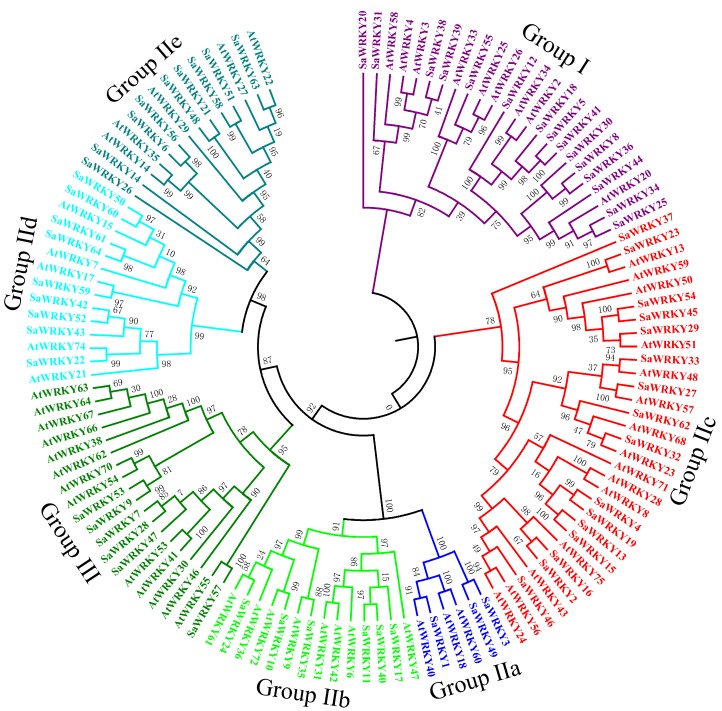
Phylogenetic tree of WRKY proteins from *Santalum album* and *Arabidopsis thaliana*. The 64 SaWRKY proteins and representative Arabidopsis WRKY proteins were aligned by ClustalX 2.0, and the NJ tree was constructed using MEGA6.0 with 1000 bootstrap replicates.

To understand the evolutionary relationship between SaWRKY proteins, the 64 identified SaWRKY proteins was examined based on AtWRKY proteins from the three groups, and an unrooted tree was built by MEGA6.0 software using the NJ method (Figure 1). All 64 SaWRKY proteins were classified into three major groups (I, II and III) (Table 1, Figure 1). Among the 15 SaWRKY proteins in group I, 13 contained two WRKY domains and the remaining two SaWRKY proteins (SaWRKY8 and 25) contained only one WRKY domain (Table 1). There were 43 SaWRKY proteins with only one WRKY domain in group II, and these could be further divided into an additional five subgroups, i.e., IIa, IIb, IIc, IId, and IIe. SaWRKY7, 9, 28, 47, 53 and 57, which contained only one WRKY domain, formed group III (Table 1).

### 2.3. Exon-Intron Organization of SaWRKY Genes

**Figure 2:**
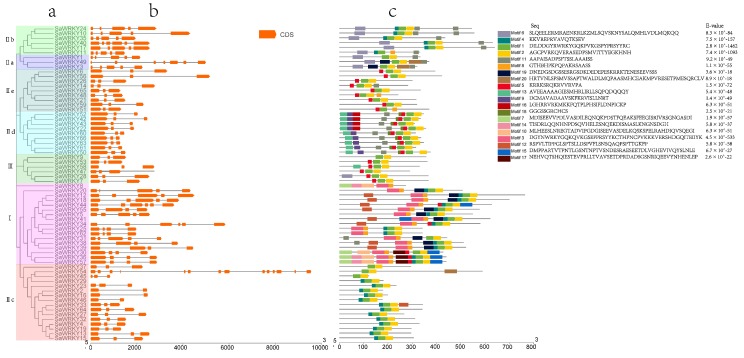
Schematic diagram showing the phylogenetic relationship among conserved motifs of SaWRKY proteins and gene structure of *SaWRKY* genes. The phylogenetic tree was built on the basis of the full amino acids of 64 SaWRKY proteins whose conserved motifs were identified using MEME.

To further understand the pivotal role that exon-intron structural features play in the evolution of *S. album* gene families, the structure of *SaWRKY* genes was obtained through exon-intron organization analysis. Among the 64 *SaWRKY* genes, three had one intron and two exons, 35 had two introns and three exons, six had three introns and four exons, nine had four introns and five exons, two had five introns and six exons, three had five introns and five exons, while the remaining *SaWRKY49* had six introns and seven exons, and *SaWRKY54* had 13 introns and 14 exons (Figure 2b). Of note, *SaWRKY* genes in the same subgroup had a similar intron and exon composition, such as two introns and three exons in group II, subgroups IId, IIe and group III, or four introns and five exons in subgroup IIb. *SaWRKY54* had 14 exons, *SaWRKY49* had seven exons, and *SaWRKY2* and *SaWRKY16* had only two exons. This indicates an occurrence of both exon gain and loss during evolution of the *SaWRKY* gene family, thus leading to functional diversity among *SaWRKY* genes.

### 2.4. Motif Composition of SaWRKY Proteins

To gain insight into the functional regions of SaWRKY proteins, the MEME program was used to predict the composition of the 64 SaWRKY protein motifs. A total of 20 conserved motifs were detected (Figure 2c). Among them, motifs 1 and 3 contained the heptapeptide stretch WRKYGQK while all 64 SaWRKY proteins contained one or two WRKYGQK motifs. Motif 2 was the conserved zinc-finger structure at the C-terminal end and was found in 61 SaWRKY proteins, but not in SaWRKY8, 11 and 51 (Figure 2). Motif 9 was unique to all members of subgroup IId, motif 16 was unique to SaWRKY42, 43 and 52, and motif 17 was unique to SaWRKY30 and 36. Similar motif compositions were found in the same groups, especially in the same subgroups, such as in subgroups IIa or IIb.

### 2.5. Prediction and Functional Enrichment Analysis of Potential SaWRKY Target Genes

**Figure 3:**
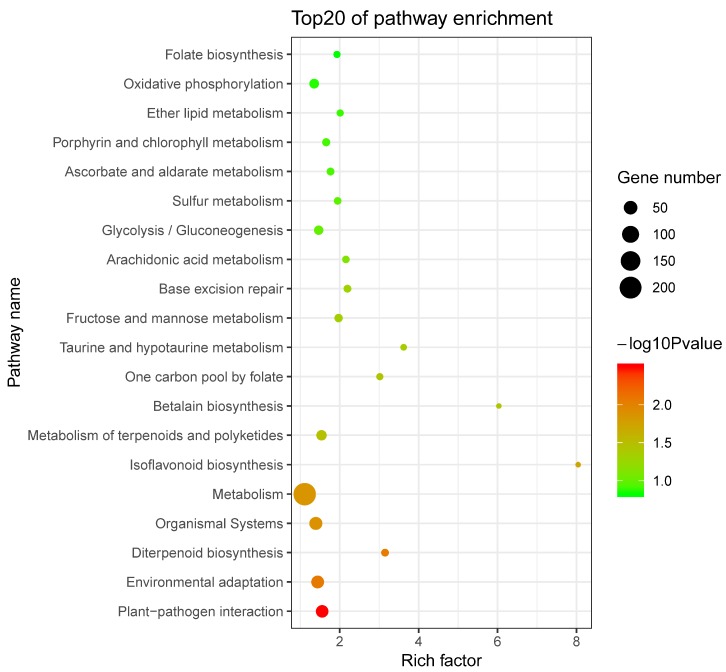
The 20 top Kyoto Encyclopedia of Genes and Genomes (KEGG) enrichment pathways of potential *SaWRKY* target genes with at least five W-boxes.

A total of 13,306 genes that contained at least one W-box in their putative promoters were identified in the assembled *S. album* genome. The number of genes decreased as more W-boxes were identified. Among all 13,306 genes, 2563 genes contained one W-box, 3007 genes contained two W-boxes, while 1328 genes contained at least five W-boxes and were used for further pathway enrichment analysis using the Kyoto Encyclopedia of Genes and Genomes (KEGG) database. As shown in Figure 3, the top enriched KEGG pathways included plant-pathogen interactions, environmental adaption, metabolism and organismal systems. These results indicate that *SaWRKY* genes are closely involved in biotic and abiotic stress responses, as well as in other biological pathways.

### 2.6. Expression Patterns of SaWRKY Genes in Different Tissues

**Figure 4:**
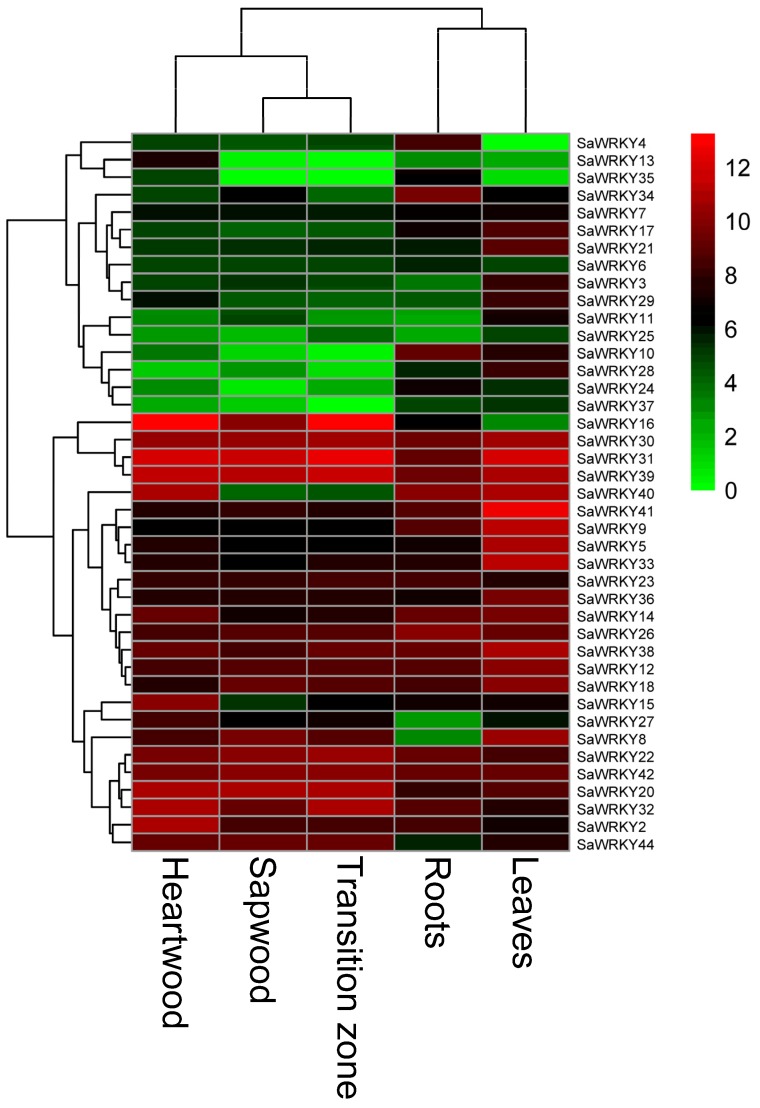
The expression patterns of 41 *SaWRKY* genes in five tissues of *Santalum album*. The heat map was generated based on the number of reads per kilobase of exon modelled per million mapped reads (RPKM) of transcriptome data.

A gene expression pattern may reflect its biological function. To explore the possible functions of *SaWRKY* genes in *S. album* development, the expression patterns of 41 *SaWRKY* genes in various tissues (leaves, roots, heartwood, sapwood, and transition zone) were obtained from the transcriptome data (Figure 4). Five *SaWRKY* genes (*SaWRKY16*, *20*, *22*, *3,* and *44*) showed a higher expression level in wood tissue (heartwood, sapwood, and transition zone) than in leaves and roots. The expression of *SaWRKY2*, *15* and *27* occurred preferentially in heartwood. Higher levels of mRNA were observed in roots for *SaWRKY4*, *10*, *26* and *34* while 12 *SaWRKY* genes (*SaWRKY3*, *5*, *8*, *9*, *17*, *21*, *28*, *29*, *33*, *36*, *38*, and *41*) showed a higher level of expression in leaves than in other tissues. *SaWRKY6*, *25*, *35* and *37* had a low level of expression in all the tissues examined, while *SaWRKY12*, *30*, *31*, *39* and *42* had consistently high expression levels in all tissues (Figure 4). It was not possible to assess the expression level of 23 *SaWRKY* genes in all tissues from the transcriptome data.

### 2.7. Expression Profiles of SaWRKY Genes in Response to SA and MeJA

**Figure 5:**
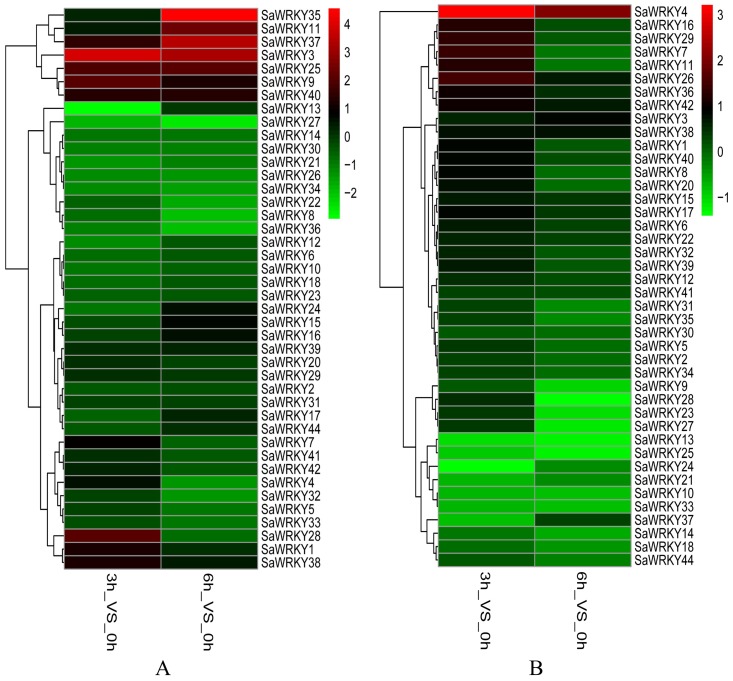
Expression profiles of *SaWRKY* genes following SA and MeJA treatments. The expression of 42 *SaWRKY* genes was determined under SA (**A**) and MeJA (**B**) stimuli at three time points (0, 3, and 6 h) in callus. The heat map was generated based on log2-transformed count value from RT-qPCR data using R language. RT-qPCR data were collected from three biological replicates.

To detect whether the *SaWRKY* genes were induced by different hormones, RT-qPCR was performed to determine the expression levels of the 42 *SaWRKY* genes when stimulated by SA and MeJA in callus. The data shows that 13 out of 42 *SaWRKY* genes were up-regulated by SA, namely *SaWRKY1*, *3*, 7, *9*, *11*, *15*, *24*, *25*, *28*, *35*, *37*, *38* and *40* (Figure 5A, Table S2). In contrast, 12 *SaWRKY* genes were down-regulated by SA: *SaWRKY4*, *8*, *12*, *13*, *21*, *22*, *26*, *27*, *30*, *32*, *34* and *36*. Several *SaWRKY* genes were regulated by MeJA. Among them, *SaWRKY1*, *3*, *4*, *6, 7*, *8*, *11*, *15*, *16*, *17*, *20*, *26*, *29*, *36*, *38*, *39*, *40* and *42* were up-regulated, whereas, *SaWRKY13*, *23*, *24*, *25*, *27* and *28* were down-regulated (Figure 5B, Table S3). Interestingly, seven *SaWRKY* genes (*SaWRKY1*, *3*, *7, 11*, *15*, *38* and *40*) were up-regulated both by SA and MeJA.

### 2.8. Characterization, Tissue Expression Patterns and Subcellular Localization of SaWRKY1

As indicated above, the expression level of *SaWRKY1* was not detected in five tissues from the transcriptome data. More interestingly, since *SaWRKY1* is one of the genes that was up-regulated both by SA and MeJA, it was selected for further analysis. The full length of the coding nucleotide sequence of *SaWRKY1* was 999 bp, encoding a protein of 332 amino acid residues with a predicted theoretical isoelectric point and protein molecular weight of 7.55 and 37.01 kD, respectively.

**Figure 6:**
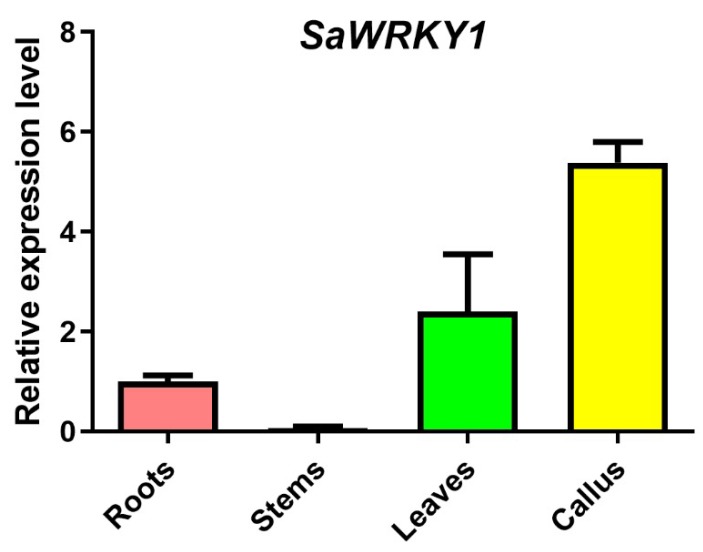
Expression of *SaWRKY1* in different tissues of *Santalum album*. Relative expression levels of *SaWRKY1* in four tissues were determined by RT-qPCR with three replicates. Values shown are means ± SE.

Tissue expression patterns, which were examined by RT-qPCR, showed that transcription of *SaWRKY1* took place mainly in callus, leaves, and roots (Figure 6). The abundance of *SaWRKY1* mRNA accumulated preferentially in callus (Figure 6).

**Figure 7:**
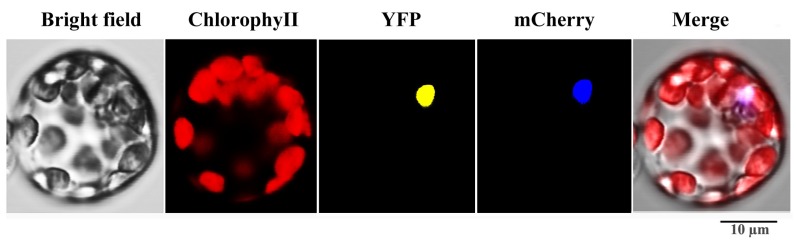
Subcellular localization assay of the SaWRKY1 protein.

The subcellular localization of the SaWRKY1 protein is most likely in the nucleus. To verify its subcellular location, a C-terminal YFP fusion construct for the SaWRKY1 protein and a nuclear location protein mCherry was co-transformed into *Arabidopsis* mesophyll protoplasts. The SaWRKY1-YFP fusion protein was localized in the nucleus and co-localized with mCherry (Figure 7), demonstrating that the SaWRKY1 protein is located in the nucleus, in accordance with its putative function as a TF.

### 2.9. Overexpression of SaWRKY1 Enhances Salinity Tolerance in Transgenic Arabidopsis Plants

*SaWRKY1* was significantly induced both by SA and MeJA, indicating that it might be a node of convergence in signal transduction pathways mediated by SA and MeJA. To further explore the roles of *SaWRKY1* in abiotic stress responses, the *35S: SaWRKY1: pCAMBIA1302* construct was transformed into *A. thaliana*, and two independent T3 homozygous progeny lines were used for further analysis.

**Figure 8:**
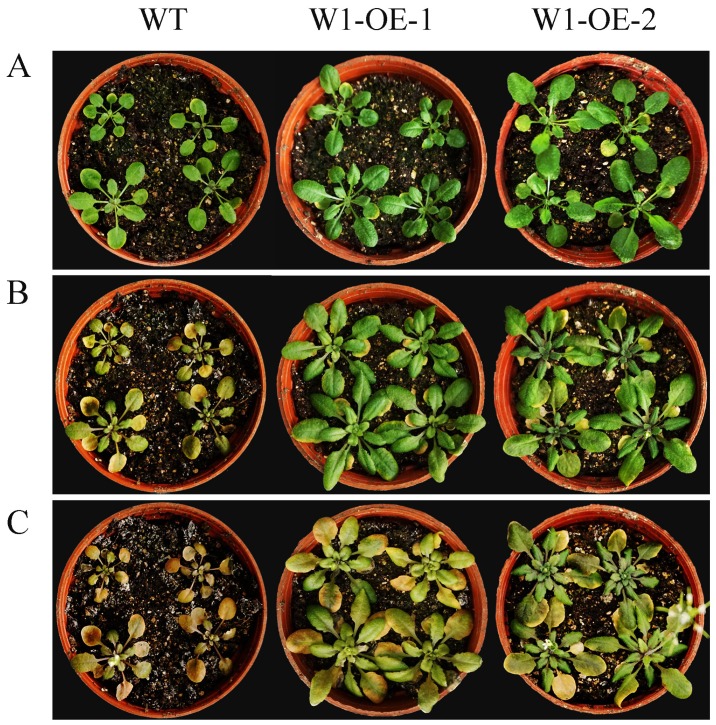
Phenotypes of two lines of overexpression *SaWRKY1* transgenic plants under salt stress. (**A**) Day 0. (**B**) Day 3 after irrigation with 300 mM NaCl. (**C**) Day 5 after second irrigation with 300 mM NaCl.

**Figure 9:**
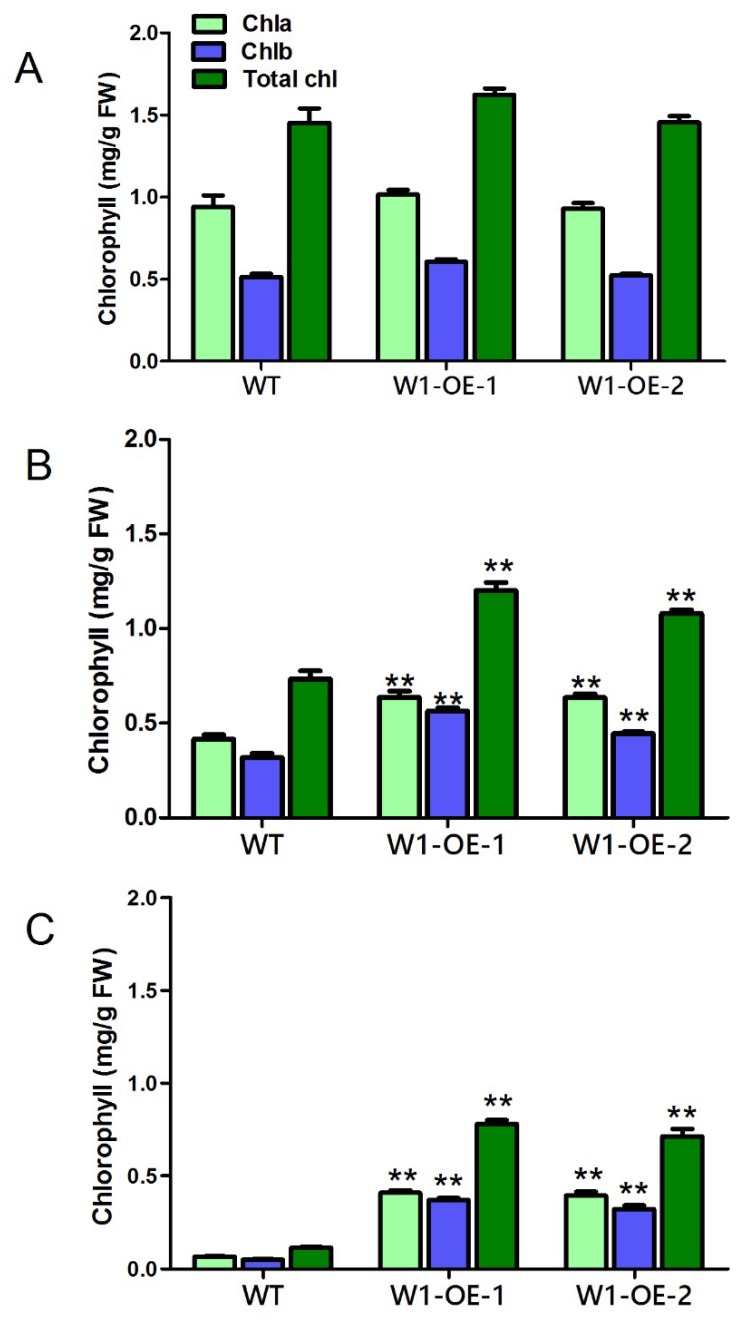
The chlorophyll content of two lines of overexpression *SaWRKY1* transgenic plants under salt stress. All values are means ± SE from at least three independent biological replicates (8 seedlings per replicate). Double asterisk (**) indicates significance at *p* < 0.01 for each component between wild type and transgenic lines using Duncan’s multiple range test. (**A**) Day 0. (**B**) Day 3 after irrigation with 300 mM NaCl. (**C**) Day 5 after second irrigation with 300 mM NaCl.

In order to examine the role of *SaWRKY1* in abiotic stress, the two *A. thaliana* transgenic lines, which showed the same level of germination, growth properties, and chlorophyll content (Figure 8A and Figure 9A), were randomly selected and irrigated with 300 mM NaCl. After three days, both transgenic lines grew well but the wild type control showed obvious inhibited growth (Figure 8A,B; Figure 9A,B). The content of chlorophyll *a*, *b*, and *a* + *b* in the wild type declined by 34.6%, 36.1% and 35.5%, respectively compared with the content of each component averaged over the two transgenic lines (Figure 8B and Figure 9B). When irrigated once again with 300 mM NaCl, unlike the wild type plants which withered and nearly died, the *35S:WRKY1* transgenic lines showed little withering, had greener leaves and slightly inhibited growth after 5 days (Figure 8A–C). Accordingly, either the content of chlorophyll *a* and *b*, or the total chlorophyll content was reduced by more than 80% in wild type *A. thaliana* compared to the content of each component in *35S:WRKY1* transgenic lines (Figure 8C and Figure 9C).

## 3. Discussion

As one of the largest TF families in flowering plants, the *WRKY* gene family participates in plant development as well as in response to abiotic and biotic stresses. Based on the *S. album* genome and transcriptome data, 64 *SaWRKY* genes were identified in our work, accounting for 100% of the *S. album* WRKY family genes that were determined by Mahesh et al. [30] using the sandalwood genome. A total of five *SaWRKY* genes (*SaWRKY1*, *19*, *43*, *45*, and *64*) were only found in the genome data but lacked transcriptome data sets, either because these genes might have a lower level of expression in some tissues while others might be pseudogenes. Three *SaWRKY* genes (*SaWRKY8*, *29* and *41*) was only found in transcriptome data, which may be caused by incomplete genome assembling or alternative splicing. The 64 SaWRKY proteins identified could be divided into three groups based on a phylogenetic analysis for *A. thaliana* and *S. album*. Group II, which contained 43 SaWRKY proteins, was further divided into five subgroups (IIa, IIb, IIc, IId, and IIe), accounting for the largest proportion (67%) among all the groups. Other plant species showed a comparable proportion, namely 58% in *A. thaliana* [6], 64% in *Caragana intermedia* [31], and 66% in cassava (*Manihot esculenta*) [32]. Chimeric proteins such as R protein-WRKY (RW) genes are a feature of some WRKY family genes in switchgrass (*Panicum virgatum*), sorghum (*Sorghum bicolor*) and rice (*Oryza sativa*) [11]. We searched for 64 SaWRKY proteins, and no other domains except for WDs and zinc-finger motifs were found. This suggest that RW genes may be a characteristic of some monocot genomes.

Publicly available transcriptome datasets were processed to obtain the RPKM expression values for the 41 *SaWRKY* genes in five different tissues of *S. album*. The heat map (Figure 4) generated using these expression values exhibited different expression patterns for all the identified *SaWRKY* genes except for *SaWRKY1* in the five tissues, indicating that they may perform diversified functions in plant development or in response to stress. *SaWRKY12*, *30*, *31*, *39*, and *42* shared similarly high expression levels in all the tested tissues (Figure 4), indicating that these genes may play essential roles in *S. album* tissue development. In contrast, four *SaWRKY* genes (*SaWRKY6*, *25*, *35*, and *37*) showed a low level of expression in all five tissues and may thus have a limited function in their development. Several *SaWRKY* genes that were identified showed higher tissue-specific expression patterns, i.e., *SaWRKY2*, *15*, *20*, *22*, *27*, *32* and *44*, which were preferentially expressed in wood tissue (sapwood, transition zone and heartwood) (Figure 4). In particular, four of these genes (*SaWRKY16*, *20*, *22*, and *32*) accumulated obviously higher levels of mRNA in the transition zone, which is the main location of the synthesis of valuable sandal essential oil [21]. For now, it can be speculated that several of these genes may be involved in essential oil biosynthesis, although this will require extensive additional analyses. In addition, RT-qPCR was performed to detect the levels of *SaWRKY1* expression in roots, stems (wood tissue), leaves and callus (Figure 6). *SaWRKY1* mRNA mainly accumulated in the leaves, roots and callus, showing the highest expression level in fast-growing tissue (i.e., callus).

Due to their sessile lifestyle, plants must adjust to various biotic and abiotic stresses [33]. Phytohormones play a central role in a plant’s ability to integrate various environmental stimuli or cues with the genetic program in their life cycles [34]. Among several phytohormones, JA and SA play crucial roles in orchestrating different stress responses within a plant, such as in pathogen defense [35], or protection against abiotic stresses (drought, low temperature, and salinity) [36]. Increasing evidence shows that the expression levels of plant *WRKY* genes are regulated by different plant hormones, such as 37 *VvWRKY* genes that were induced by SA, and 48 *VvWRKY* genes that were regulated following JA treatment in grape (*Vitis vinifera*) [37], or 55 *PtrWRKY* genes that were induced by SA and MeJA treatment in poplar (*Populus trichocarpa* and *P. tomentosa*) [9]. It is difficult to conduct basic research in woody plants because of their slow growth, so callus is a useful and efficient system to conduct gene function research on plants such as oil palm (*Elaeis guineensis*) [38] and apple (*Malus x domestica*) [39]. In order to detect the expression levels of the *SaWRKY* genes in response to SA and MeJA, RT-qPCR was performed in callus generated from newly sprouted *S. album* shoots. RT-qPCR results showed 13 up-regulated and 12 down-regulated *SaWRKY* genes after SA treatment (Figure 5A, Table S2), and 18 up-regulated and six down-regulated *SaWRKY* genes after JA treatment (Figure 5B, Table S3). This indicates that these genes may be involved in biotic and abiotic stress responses mediated by SA- and/or JA-dependent defense signaling pathways. *AtWRKY53*, a paralogous gene of *SaWRKY7* and *SaWRKY28* in *S. album*, was significantly induced in five- and seven-week-old *A. thaliana* plants sprayed with 2 mM SA [40]. *AtWRKY53* expression was up-regulated after pathogen attack [41], and this gene participates in the regulation of senescence, being positively regulated by SA signaling and negatively regulated by JA signaling [40,42], and negatively regulates drought tolerance via controlled stomatal movement independent of abscisic acid levels [43]. It can thus be inferred that paralogous genes *SaWRKY7* and *28* may perform a similar function in *S. album*. A member of subgroup IIc, *SaWRKY16*, which showed the highest level of expression in the transition zone of wood tissue, was significantly induced by JA (Figure 5B, Table S3). Its paralogous gene, *AtWRKY75*, played a positive role in the defense of *A. thaliana* against pathogens (including *Pectobacterium carotovorum* ssp. *carotovorum* and *Sclerotinia sclerotiorum*) and oxalic acid stress, and was mainly dependent on the JA/ethylene pathway [44,45]. Two *SaWRKY* genes (*SaWRKY25* and *28*) were up-regulated by SA but down-regulated by JA (Figure 5A,B; Tables S2 and S3), consistent with some reports showing that SA and JA function antagonistically [46,47,48]. Conversely, seven *SaWRKY* genes, *SaWRKY1*, *3*, *7*, *11*, *15*, *38* and *40*, were up-regulated when stimulated by SA and JA. *SaWRKY1* was one of the genes induced both by SA and JA treatment, and its overexpression strengthened salt tolerance and increased the content of chlorophyll *a*, *b*, and *a* + *b* in transgenic *A. thaliana* plants compared to the wild type. Plants often enhance the content of non-enzymatic compounds such as chlorophyll to improve tolerance to salt stress, and a rich literature on this topic illustrates that SA and JA perform important roles in these processes. For example, strawberry (*Fragaria* × *ananassa*) plants treated with 1.0 mM SA had a higher chlorophyll content and showed better growth under salt stress than unstressed plants [49]. The application of 0.01 mM SA to tomato (*Solanum lycopersicum*) plants caused the accumulation of photosynthetic pigments and soluble sugars, and enhanced plant growth and development under salt stress [50]. Foliar application of 2.0 mM JA to wheat seedlings enhanced their salt stress tolerance and growth by increasing the activity of antioxidant enzymes and the content of glutathione, chlorophyll *b* and carotenoids [51]. Cumulatively, these findings coincide with our results and suggest that *SaWRKY1* improves plant salt tolerance by increasing the content of photosynthetic pigments, probably mediated by SA and/or JA. However, the accurate regulatory mechanism still needs to be studied in further detail. In *A. thaliana*, the paralogous genes of *SaWRKY1*, *3* and *49*, namely *AtWRKY18*, *40* and *60*, are evolutionarily closely related WRKY domain TFs. Based on multiple alignments of amino acid sequences (Figure S1), there was 36.02%, 40.82% and 33.87% identity between *SaWRKY1* and *AtWRKY18*, *40*, and *60*, respectively. *SaWRKY1* has the closest evolutionary relationship and highest amino acid sequence identity with *AtWRKY40*, which is also involved in salt stress [52], suggesting that these paralogous genes play similar roles in plants against abiotic stress and confirming that *SaWRKY1* plays a regulatory role in salt resistance. Thus, *SaWRKY1* may be useful in engineering salinity tolerance of *S. album* trees via *Agrobacterium*- or gene gun-mediated transformation of callus. As two members of subgroup IIa, *SaWRKY3* and *SaWRKY49* might also be involved in SA- and JA-mediated pathogen and salt stress responses, but the putative function needs to be further investigated.

## 4. Materials and Methods

### 4.1. Plant Materials and Hormone Treatments

The leaves, roots and stems were collected from three seven-year-old *S. album* trees growing in South China Botanical Garden. Callus was induced from newly sprouted shoots following previously reported methods [25,53], with a few modifications, as described next. Shoot segments were inoculated on solid Murashige and Skoog (MS) basal medium [54] to which 1.0 mg/L thidiazuron (TDZ) was added to induce callus. The resulting callus was proliferated on solid MS medium supplemented with 1.5 mg/L 2,4-dichlorophenoxyacetic acid (2,4-D) and 0.2 mg/L TDZ. After about 20 days, similarly good callus (Figure S2) was proliferated after transferring into liquid MS basal medium for 20 days then shaken continuously at 100 rpm under the same culture conditions. After 24 h, SA and MeJA were added separately at a final concentration of 100 µM each. Samples were collected at 0, 3 and 6 h with three biological triplicates per treatment. All samples were frozen immediately in liquid nitrogen (N2) and stored at −80 °C until use.

### 4.2. Identification of WRKY Genes in S. album and Prediction of Subcellular Localization

The *S. album* genome was downloaded from the National Center for Biotechnology Information (NCBI) (GCA_002925775.1) and then annotated using sandalwood transcriptome sequencing data and genome sequence data from maize, rice and Arabidopsis by a web service for training AUGUSTUS and predicting genes in eukaryotes (WebAUGUSTUS; https://bio.tools/webaugustus). *S. album* transcriptome sequencing data was downloaded from the NCBI Sequence Read Archive (SRA) (GenBank accessions: PRJNA297453, SRA150639, SRR3731808, and SRR3731809), and the *AtWRKY* gene sequences were searched and downloaded from The Arabidopsis Information Resource (TAIR) website (http://www.arabidopsis.org/index.jsp). All the *S. album WRKY* genes were identified following He et al. [55]. In brief, candidate *SaWRKY* genes were obtained by searching *S. album* protein sequences using the hidden Markov model (http://hmmer.janelia.org/). Putative *SaWRKY* genes were further verified for the presence of the PFAM domain (PF03106) in the pFAM database (http://pfam.xfam.org/) and then screened for the WRKY motif and zinc-finger domains, and finally confirmed as *SaWRKY* genes after removing redundant sequences by CD-HIT software (see Table S1). The subcellular localization of *SaWRKY* genes was predicted using the PSORT program (https://psort.hgc.jp/).

### 4.3. Construction of Phylogenetic Tree and Conserved Motif Analysis of SaWRKY Proteins

An unrooted phylogenetic tree of *S. album* and Arabidopsis WRKY proteins was constructed using MEGA6.0 software with 1000 bootstrap replicates. The parameters used to construct trees were: gaps/missing data: complete deletion; substitution model: maximum composite likelihood; rates among sites: gamma distributed with invariant sites (G).

The online program MEME v 4.11.2 (http://meme.nbcr.net/meme/) was used to analyze conserved motifs for the 64 *S. album* WRKY protein sequences. MEME parameters were set as advised by He et al. [55]. The TBtool was used to draw a schematic diagram of the conserved motifs and gene structures.

### 4.4. Identification and Functional Enrichment Analysis of Potential SaWRKY Target Genes

The 2-k DNA sequence upstream of the ATG start codon of each gene assembled from the *S. album* genome was used to scan the WRKY TF binding site element with the sequence (C/T)TGAC(C/T). The *SaWRKY* genes with at least five WRKY binding sites were identified as potential *SaWRKY* target genes, and were used for further pathway enrichment analysis using the KEGG database. The top 20 enrichment KEGG pathways were drawn with R package ggplot2.

### 4.5. Gene Expression Analysis by Quantitative real-Time PCR (RT-qPCR)

Total RNA from *S. album* callus was isolated using the Eastep^®^ Super Total RNA Extraction kit (Promega, Shanghai, China), and the RNA from leaves, stems and roots was extracted using a protocol reported for the isolation of RNA from woody plants [56]. The quality and quantity of DNA-free total RNA was assessed using a NanoDrop ND-1000 spectrophotometer (Nanodrop Technologies, Wilmington, NC, USA). RNA samples with an A260/A280 ratio between 1.9 and 2.1 and an A260/A230 ratio greater than 2.0 were used for subsequent analysis. RNA integrity was assessed by 1.5% (*w*/*v*) agarose gel electrophoresis. Total RNA (1 µg) was used to synthesize first-strand cDNAs using an equivalent amount of oligo-(dT)15 and random primers in 20 µL volume with the GoScript™ Reverse Transcriptase system (Promega, Madison, WI, USA) according to the manufacturer’s protocols. Amplification was performed at 25 °C for 5 min, 42 °C for 60 min, and 70 °C for 15 min. The successfully synthesized cDNA samples were diluted 1:10 with nuclease-free water and stored at −20 °C until further use.

RT-qPCR was performed in 96-well plates in an ABI 7500 Real-time system (ABI, Alameda, CA, USA) using the SoAdvancedTM Universal SYBR^®^ Green Supermix detection system (Bio-Rad, Hercules, CA, USA). The RT-qPCR reaction in a total volume of 10 µL consisted of 5 µL SYBR^®^ Green Supermix, 0.5 µL of forward primer (10 µM), 0.5 µL of reverse primer (10 µM), 1 µL of cDNA, and 3 µL of ddH_2_O. The cycling conditions were 95 °C for 2 min, followed by 40 cycles at 95 °C for 15 s and 60 °C for 1 min. After 40 cycles, melting curve analysis was performed ranging from 60 to 95 °C. The internal reference genes used in different tissues and phytohormone treatments followed Yan et al. [57], i.e., *FAB1A* and *PP2C* for four tissues, *ODD* and *Fbp1* for the SA treatment, as well as *CSA* and *Fbp3* for the MeJA treatment. The relative gene expression level was calculated using the 2^−ΔΔ*C*q^ method. All RT-qPCR experiments were carried out using three biological replicates of each sample. The gene-specific primers used for RT-qPCR are listed in Table S4.

### 4.6. Cluster Analysis of Expression Data

For the expression profiles of all putative *SaWRKY* genes in different tissues including leaves, roots and wood (containing heartwood, sapwood and the transition zone), the data that was obtained from the NCBI SRA database was mapped with *SaWRKY* gene nucleotide sequences using TopHat version 2.0.8. The level of gene expression was calculated by reads per kilobase of exon model per million mapped reads (RPKM), and the expression profiles via a heat map were calculated from the log2 (FPKM+1) value, and shown as a green-black-red gradient by using the heat map package in R version 3.4.0. Expression (*C*q) values of the 42 putative *SaWRKY* genes at each time point in SA and MeJA treatments were determined using RT-qPCR. The expression profiles were generated by a heat map using R language based on the log2 (2^−ΔΔ*C*q^) value. Up- and down-regulated genes were defined as those showing more than a 1.5-fold change or lower than a 0.5-fold change, respectively (*p* < 0.05).

### 4.7. Cloning and Subcellular Localization of SaWRKY1

RACE was performed to obtain the full-length sequence of *SaWRKY1* using the SMARTer RACE cDNA Amplification Kit (Takara Bio USA, Inc., Mountain View, CA, USA; see Method S1, Figure S3). The amplification product was sequenced at the Beijing Genomics Institute (BGI, Shenzhen, China), and then submitted to NCBI (accession number: MG655189). The theoretical isoelectric point and protein molecular weight were determined using ExPASy server (https://web.expasy.org/cgi-bin/protparam/protparam).

The full-length coding sequence of *SaWRKY1* without the termination codon was amplified with gene-specific primers (SaWRKY1YFPF, 5′-GAACGATAGCCATGGCAATGGATTACTCTTCTTGGAC-3′; SaWRKY1YFPR, 5′-TGAGTCCGGACCATGGTAAATTTTTCATTTTGACTTT-3′) and then fused to yellow fluorescent protein (YFP) behind the Cauliflower mosaic virus (CaMV) 35S promoter in the *Nco*I site of the pSAT6-EYFP-N1 vector following the In-Fusion HD Cloning Kit (Clontech) instructions. The *35S: SaWRKY1: pSAT6-EYFP-N1* construct was confirmed by sequencing and then co-transformed with a nuclear location protein mCherry into *A. thaliana* mesophyll protoplasts according to a protocol described by Yoo et al. [58]. After 12 h incubation at 22 °C under light conditions, YFP fluorescence was visualized with a Zeiss LSM 510 confocal microscope (Zeiss, Jena, Germany), and images were captured using the same software.

### 4.8. Arabidopsis thaliana Transformation and Salinity Stress

The full-length coding sequence of *SaWRKY1* was amplified with gene-specific primers (SaWRKY1OXF, 5′-GGACTCTTGACCATGGTAATGGATTACTCTTCTTGGACGGC-3′; SaWRKY1OXR, 5′-GTCAGATCTACCATGGTAAATTTTTCATTTTGACTTT-3′) and then cloned into the pCAMBIA1302 vector behind the CaMV 35S promoter in the *Nco*I site using the In-Fusion HD Cloning Kit. The resulting *35S: SaWRKY1: pCAMBIA1302* construct, which was confirmed by sequencing and then transformed into *Agrobacterium tumefaciens* EHA105 using the freeze-thaw method described by Weigel and Glazebrook [59], was introduced into *A. thaliana* by the floral dip method [60]. Seeds of the T_0_ generation were collected from transgenic Arabidopsis. Thereafter, transformants were screened on half-strength MS medium containing 25 mg/L hygromycin. A total of 28 individual hygromycin-resistant plants, which developed two main leaves and roots, were obtained. Their transgenic nature was confirmed by RT-PCR with gene-specific primers (F: 5′-ATGGATTACTCTTCTTGGACGGC-3′; R: 5′-TCAAAATTTTTCATTTTGACTTT-3′) using their own genomic DNA as template. The seeds of 26 transgenic hygromycin-resistant T_1_ generation plants containing the full-length coding sequence of *SaWRKY1* were harvested separately from each individual plant. The 26 transgenic hygromycin-resistant plants were considered as 26 independent transgenic lines, which were further screened as mentioned above separately. Eight independent transgenic lines which showed a Mendelian ratio of about 3:1 were selected to harvest T_2_ generation seeds from each plant. These eight lines were further screened as mentioned above, and two independent transgenic lines that did not segregate were selected to harvest T_3_ generation seeds from each plant. RT-qPCR was performed using gene-specific primers (SaWRKY1qPCRF/R see Table S4) to confirm the expression of *SaWRKY1* in two lines of T_3_ homozygous transformed plants (Figure S4). Two *SaWRKY1* overexpression transgenic lines from the homozygous T3 generation, which were about 4 weeks old, were irrigated with 300 mM NaCl at three-day intervals to observe their phenotypes and to determine chlorophyll content using aqueous acetone as the solvent, as described by Porra et al. [61]. On the same dates, *A. thaliana* ecotype Columbia (Col-0) was also watered with 300 mM NaCl and used as the control. Experiments were performed at least three times to observe resulting phenotypes and to determine chlorophyll content.

### 4.9. Statistical Analyses

Statistical analysis was performed using SPSS 19.0 (IBM Corp., Armonk, NY, USA). Following one-way analysis of variance (ANOVA), significance between treatment means was assessed by Duncan’s multiple range test at *p* < 0.05 and *p* < 0.01.

## 5. Conclusions

In this study, we identified 64 *SaWRKY* genes in Indian sandalwood based on genome and transcriptome data. The phylogenetic relationships, composition of conserved motifs and tissue expression patterns of these genes were analyzed. Callus was used to efficiently detect the expression profiles of 42 *SaWRKY* genes under different phytohormone treatments, suggesting that 25 and 24 *SaWRKY* genes are involved in SA- and MeJA-mediated abiotic or biotic response pathways, respectively. In addition, functional analysis of *SaWRKY1* under salt stress increased our understanding of the roles that *SaWRKY1* play in abiotic stress.

## Figures and Tables

**Table 1:** List of *SaWRKY* genes identified in *Santalum album*.

Gene Name	ORF (aa)	Conserved Motif	Zinc-Finger Type	Subcellular Location	Group
*SaWRKY5*	549	WRKYGQK/WRKYGQK	C-X_4_-C-X_22_-HXH/C-X_4_-C-X_23_-HXH	Nucleus	I
*SaWRKY8*	273	WRKYGQK	C-X_4_-C-X_22_-HXH	Golgi	I
*SaWRKY12*	764	WRKYGQK/WRKYGQK	C-X_5-_C-X_23_-HXT/C-X_4_-C-X_22_-HXH/ C-X_4_-C-X_23_-HXH	Nucleus	I
*SaWRKY18*	700	WRKYGQK/WRKYGQK	C-X_4_-C-X_22_-HXH/C-X_4_-C-X_23_-HXH	Nucleus	I
*SaWRKY20*	507	WRKYGQK/WRKYGQK	C-X_4_-C-X_22_-HXH/C-X_4_-C-X_23_-HXH	Nucleus	I
*SaWRKY25*	342	WRKYGQK	C-X_4_-C-X_22_-HXH/C-X_4_-C-X_23_-HXH/ C-X_7_-C-X_23_-HXL	Nucleus	I
*SaWRKY30*	427	WRKYGQK/WRKYGQK	C-X_4_-C-X_22_-HXH/C-X_4_-C-X_23_-HXH	Nucleus	I
*SaWRKY31*	443	WRKYGQK/WRKYGQK	C-X_4_-C-X_22_-HXH/C-X_4_-C-X_23_-HXH	Nucleus	I
*SaWRKY34*	580	WRKYGQK/WRKYGQK	C-X_4_-C-X_22_-HXH/C-X_4_-C-X_23_-HXH	Nucleus	I
*SaWRKY36*	440	WRKYGQK/WRKYGQK	C-X_4_-C-X_22_-HXH/C-X_4_-C-X_23_-HXH	Nucleus	I
*SaWRKY38*	511	WRKYGQK/WRKYGQK	C-X_4_-C-X_22_-HXH/C-X_4_-C-X_23_-HXH	Nucleus	I
*SaWRKY39*	521	WRKYGQK/WRKYGQK	C-X_4_-C-X_22_-HXH/C-X_4_-C-X_23_-HXH	Nucleus	I
*SaWRKY41*	621	WRKYGQK/WRKYGQK	C-X_4_-C-X_22_-HXH/C-X_5_-C-X_23_-HXH	Nucleus	I
*SaWRKY44*	627	WRKYGQK/WRKYGQK	C-X_4_-C-X_22_-HXH/C-X_4_-C-X_23_-HXH	Nucleus	I
*SaWRKY55*	577	WRKYGQK/WRKYGQK	C-X_4_-C-X_22_-HXH/C-X_4_-C-X_23_-HXH	Nucleus	I
*SaWRKY1*	333	WRKYGQK	C-X_5_-C-X_23_-HXH	Nucleus	IIa
*SaWRKY3*	320	WRKYGQK	C-X_5_-C-X_23_-HXH	Nucleus	IIa
*SaWRKY49*	369	WRKYGQK	C-X_5_-C-X_23_-HXH	Chloroplast	IIa
*SaWRKY10*	555	WRKYGQK	C-X_5_-C-X_23_-HXH	Nucleus	IIb
*SaWRKY11*	331	WRKYGQK	C-X_6_-CX_23_-HXH	Nucleus	IIb
*SaWRKY17*	639	WRKYGQK	C-X_5_-C-X_23_-HXH	Nucleus	IIb
*SaWRKY24*	545	WRKYGQK	C-X_5_-C-X_23_-HXH	Nucleus	IIb
*SaWRKY35*	435	WRKYGQK	C-X_5_-C-X_23_-HXH	Nucleus	IIb
*SaWRKY40*	631	WRKYGQK	C-X_5_-C-X_23_-HXH	Nucleus	IIb
*SaWRKY2*	179	WRKYGQK	C-X_4_-C-X_23_-HXH	Nucleus	IIc
*SaWRKY4*	329	WRKYGQK	C-X_4_-C-X_23_-HXH	Nucleus	IIc
*SaWRKY13*	295	WRKYGQK	C-X_4_-C-X_23_-HXH	Nucleus	IIc
*SaWRKY15*	306	WRKYGQK	C-X_4_-C-X_23_-HXH	Nucleus	IIc
*SaWRKY16*	199	WRKYGQK	C-X_4_-C-X_23_-HXH	Nucleus	IIc
*SaWRKY19*	296	WRKYGQK	C-X_4_-C-X_23_-HXH	Nucleus	IIc
*SaWRKY23*	234	WRKYGQK	C-X_4_-C-X_23_-HXH	Nucleus	IIc
*SaWRKY27*	266	WRKYGQK	C-X_4_-C-X_23_-HXH	Nucleus	IIc
*SaWRKY29*	181	WRKYGKK	C-X_4_-C-X_23_-HXH	Nucleus	IIc
*SaWRKY32*	311	WRKYGQK	C-X_4_-C-X_23_-HXH	Nucleus	IIc
*SaWRKY33*	343	WRKYGQK	C-X_4_-C-X_23_-HXH	Nucleus	IIc
*SaWRKY37*	294	WRKYGQK	C-X_4_-C-X_23_-HXH	Nucleus	IIc
*SaWRKY45*	121	WRKYGHK	C-X_4_-C-X_23_-HXY	cytoplasm	IIc
*SaWRKY46*	177	WRKYGQK	C-X_4_-C-X_23_-HXH	peroxisome	IIc
*SaWRKY54*	589	WRKYGKK	C-X_4_-C-X_23_-HXH	Nucleus	IIc
*SaWRKY62*	341	WRKYGQK	C-X4-C-X23-HXH	Nucleus	IIc
*SaWRKY22*	356	WRKYGQK	C-X_5_-C-X_23_-HXH	Nucleus	IId
*SaWRKY42*	335	WRKYGQK	C-X_4_-C-X_22_-HXV/C-X_5_-C-X_23_-HXH	Nucleus	IId
*SaWRKY43*	310	WRKYGQK	C-X_5_-C-X_23_-HXH	Nucleus	IId
*SaWRKY50*	375	WRKYGQK	C-X_5_-C-X_23_-HXH	Nucleus	IId
*SaWRKY52*	347	WRKYGQK	C-X_5_-C-X_23_-HXH	Nucleus	IId
*SaWRKY59*	314	WRKYGQK	C-X5-C-X23-HXH	Nucleus	IId
*SaWRKY60*	314	WRKYGQK	C-X_5_-C-X_23_-HXH	Nucleus	IId
*SaWRKY61*	315	WRKYGQK	C-X_5_-C-X_23_-HXH	Nucleus	IId
*SaWRKY64*	336	WRKYGQK	C-X5-C-X23-HXH	Nucleus	IId
*SaWRKY6*	394	WRKYGQK	C-X_5_-C-X_23_-HXH	Nucleus	IIe
*SaWRKY14*	282	WRKYGQK	C-X_5_-C-X_23_-HXH	Nucleus	IIe
*SaWRKY21*	316	WRKYGQK	C-X_5_-C-X_23_-HXH	Nucleus	IIe
*SaWRKY26*	300	WRKYGQK	C-X_5_-C-X_23_-HXH	Nucleus	IIe
*SaWRKY48*	242	WRKYGQK	C-X_5_-C-X_23_-HXH	Nucleus	IIe
*SaWRKY51*	369	WRKYGQK	C-X_3_-C-X_22_-HXL/C-X_5_-C-X_23_-HXH	Nucleus	IIe
*SaWRKY56*	422	WRKYGQK	C-X_5_-C-X_23_-HXH	Nucleus	IIe
*SaWRKY5* *8*	319	WRKYGQK	C-X5-C-X22-HXS	Nucleus	IIe
*SaWRKY63*	334	WRKYGQK	C-X5-C-X23-HXH	Nucleus	IIe
*SaWRKY7*	367	WRKYGQK	C-X_7_-C-X_23_-HXC	Nucleus	III
*SaWRKY9*	357	WRKYGQK	C-X_7_-C-X_23_-HXC	Nucleus	III
*SaWRKY28*	366	WRKYGQK	C-X_7_-C-X_23_-HXC	Nucleus	III
*SaWRKY47*	290	WRKYGQK	C-X_7_-C-X_23_-HXC	Nucleus	III
*SaWRKY53*	362	WRKYGQK	C-X7-C-X22-HXT	Nucleus	III
*SaWRKY57*	330	WRKYGQK	C-X_7_-C-X_23_-HXC	Nucleus	III

## Supplementary Materials

Supplementary materials can be found at https://www.mdpi.com/1422-0067/20/22/5676/s1.
