# Supplementary material for: Genome-Wide Characterization, Expression Profile Analysis of WRKY Family Genes in Santalum album and Functional Identification of Their Role in Abiotic Stress
Source: Int J Mol Sci. 2019 Nov 13;20(22):5676. doi: 10.3390/ijms20225676 (PMC6888422; doi:10.3390/ijms20225676)
Supplement: Supplementary file 1 [file ijms-20-05676-s001.pdf]

Table S1. The GenBank accession numbers of 64 *SaWRKY* genes.

Gene name	GenBank accession number	Gene name	GenBank accession number
SaWRKY1	MG655189	*SaWRKY33*	MN335843
SaWRKY2	MN335812	*SaWRKY34*	MN335844
SaWRKY3	MN335813	*SaWRKY35*	MN335845
SaWRKY4	MN335814	*SaWRKY36*	MN335846
SaWRKY5	MN335815	*SaWRKY37*	MN335847
SaWRKY6	MN335816	*SaWRKY38*	MN335848
SaWRKY7	MN335817	*SaWRKY39*	MN335849
SaWRKY8	MN335818	*SaWRKY40*	MN335850
SaWRKY9	MN335819	*SaWRKY41*	MN335851
SaWRKY10	MN335820	*SaWRKY42*	MN335852
SaWRKY11	MN335821	*SaWRKY43*	MN335853
SaWRKY12	MN335822	*SaWRKY44*	MN335854
SaWRKY13	MN335823	*SaWRKY45*	MN335855
SaWRKY14	MN335824	*SaWRKY46*	MN335856
SaWRKY15	MN335825	*SaWRKY47*	MN335857
SaWRKY16	MN335826	*SaWRKY48*	MN335858
SaWRKY17	MN335827	*SaWRKY49*	MN335859
SaWRKY18	MN335828	*SaWRKY50*	MN335860
SaWRKY19	MN335829	*SaWRKY51*	MN335861
SaWRKY20	MN335830	*SaWRKY52*	MN335862
SaWRKY21	MN335831	*SaWRKY53*	MN335863
SaWRKY22	MN335832	*SaWRKY54*	MN335864
SaWRKY23	MN335833	*SaWRKY55*	MN335865
SaWRKY24	MN335834	*SaWRKY56*	MN335866
SaWRKY25	MN335835	*SaWRKY57*	MN335867
SaWRKY26	MN335836	*SaWRKY58*	MN335868
SaWRKY27	MN335837	*SaWRKY59*	MN335869
SaWRKY28	MN335838	*SaWRKY60*	MN335870
SaWRKY29	MN335839	*SaWRKY61*	MN335871
SaWRKY30	MN335840	*SaWRKY62*	MN335872
SaWRKY31	MN335841	*SaWRKY63*	MN335873
SaWRKY32	MN335842	*SaWRKY64*	MN335874

Table S2. The fold change of *SaWRKY* genes under SA stress in callus of *S. album* calculated from the $2^{-\Delta\Delta Cq}$ value.

Gene name	3 h		6 h	
	fold change	regulation	fold change	regulation
SaWRKY1	2.206797275	up	1.14750389	
SaWRKY2	0.738134085		0.819406687	
SaWRKY3	14.90117762	up	9.509590983	up
SaWRKY4	1.472105145		0.39267108	down
SaWRKY5	0.929858303		0.548151006	
SaWRKY6	0.571152386		0.726327684	
SaWRKY7	1.813481997	up	0.691940304	
SaWRKY8	0.566519826		0.253004586	down
SaWRKY9	4.178668397	up	2.31997935	up
SaWRKY10	0.546480355		0.691907677	
SaWRKY11	1.420085027		5.212053281	up
SaWRKY12	0.442549493	down	0.744507345	
SaWRKY13	0.131955327	down	0.980030335	
SaWRKY14	0.521100051		0.553634974	
SaWRKY15	0.801186024		1.720169597	up
SaWRKY16	0.887492474		1.49123034	
SaWRKY17	0.635988087		1.198649418	
SaWRKY18	0.619998824		0.747507923	
SaWRKY20	1.110148034		0.91190098	
SaWRKY21	0.387679448	down	0.459665935	down
SaWRKY22	0.645768995		0.333305932	down
SaWRKY23	0.674368844		0.750873586	
SaWRKY24	0.51170785		1.551569184	up
SaWRKY25	3.867605091	up	4.482831502	up
SaWRKY26	0.440543513	down	0.400721111	down
SaWRKY27	0.274681837	down	0.165934562	down
SaWRKY28	4.173614691	up	0.608460515	
SaWRKY29	1.128701394		0.963280002	
SaWRKY30	0.47464644	down	0.481256398	down
SaWRKY31	0.863530447		0.909956815	
SaWRKY32	0.915718443		0.38254082	down
SaWRKY33	0.822791255		0.510075435	
SaWRKY34	0.446491486	down	0.371755134	down
SaWRKY35	1.174505475		23.57493436	up
SaWRKY36	0.468041766	down	0.251033015	down
SaWRKY37	2.686446144	up	10.67282853	up
SaWRKY38	2.26204041	up	1.362718363	
SaWRKY39	1.102451328		1.165597215	

SaWRKY40	2.48301904	up	2.642286316	up
SaWRKY41	1.113994177		0.694815365	
SaWRKY42	1.167922597		0.752770731	
SaWRKY44	0.764665187		1.148625057	

Table S3. The fold change of *SaWRKY* genes under MeJA stress in callus of *S. album* calculated from the $2^{-\Delta\Delta Cq}$ value.

Gene name	3 h		6 h	
	fold change	regulation	fold change	regulation
SaWRKY1	1.850499998	up	1.116481287	
SaWRKY2	1.234830365		0.944131409	
SaWRKY3	1.460169714		1.793155605	up
SaWRKY4	9.291316934	up	4.04942672	up
SaWRKY5	1.295365194		0.980524758	
SaWRKY6	1.554448956	up	1.221890726	
SaWRKY7	2.648604789	up	0.920299045	
SaWRKY8	1.863783813	up	0.948244669	
SaWRKY9	1.055996357		0.519400591	
SaWRKY10	0.598666561		0.558012883	
SaWRKY11	2.396926724	up	0.882103176	
SaWRKY12	1.366054073		1.153669389	
SaWRKY13	0.467230263	down	0.409969384	down
SaWRKY14	0.883029921		0.666614508	
SaWRKY15	1.587356106	up	1.319549139	
SaWRKY16	2.320418765	up	1.127707512	
SaWRKY17	1.769904088	up	1.303084813	
SaWRKY18	0.958319167		0.761902059	
SaWRKY20	1.675082345	up	0.973615387	
SaWRKY21	0.627552569		0.7860783	
SaWRKY22	1.481163575		1.203111244	
SaWRKY23	1.304516794		0.468479142	down
SaWRKY24	0.378559663	down	0.768066635	
SaWRKY25	0.530062154		0.412450775	down
SaWRKY26	2.804674798	up	1.615387795	up
SaWRKY27	1.294998757		0.448508441	down
SaWRKY28	1.38483034		0.398180941	down
SaWRKY29	2.486928841	up	1.061897949	
SaWRKY30	1.078970351		0.973538159	
SaWRKY31	1.258091073		0.787537378	
SaWRKY32	1.486242714		1.121918055	
SaWRKY33	0.630597355		0.58252386	
SaWRKY34	1.205765567		0.933522999	
SaWRKY35	1.215475927		0.783541428	
SaWRKY36	2.052148714	up	1.381840289	
SaWRKY37	0.587130922		1.220275288	
SaWRKY38	1.661171585	up	1.757088241	up
SaWRKY39	1.548007285	up	1.08405258	

SaWRKY40	1.781933089	up	1.16922189	
SaWRKY41	1.215749675		1.193757678	
SaWRKY42	2.016003264	up	1.58294975	up
SaWRKY44	1.064059237		0.837382937	

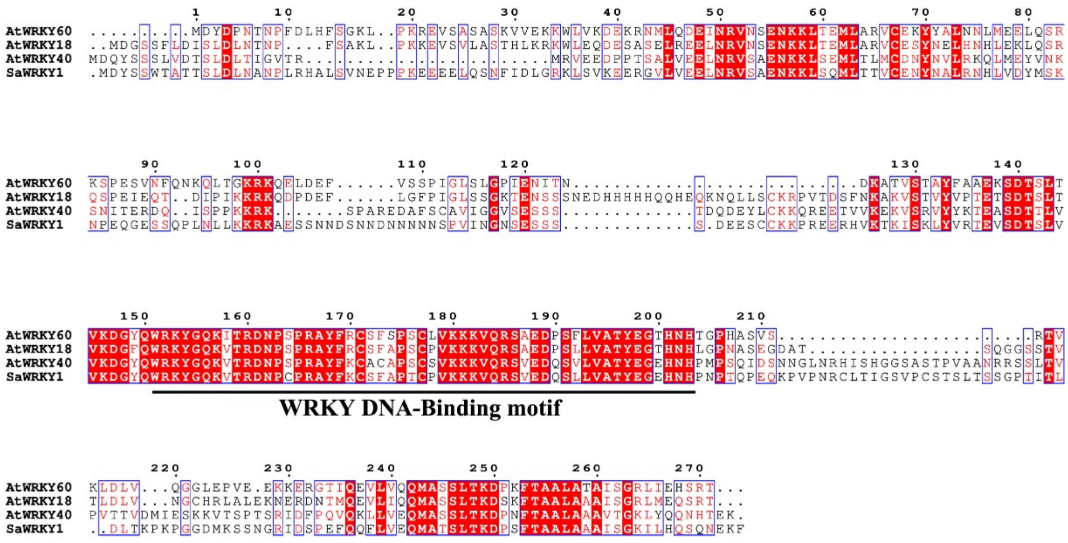

Figure S1. Multiple sequence alignment among SaWRKY1, AtWRKY40, AtWRKY18, and AtWRKY60.

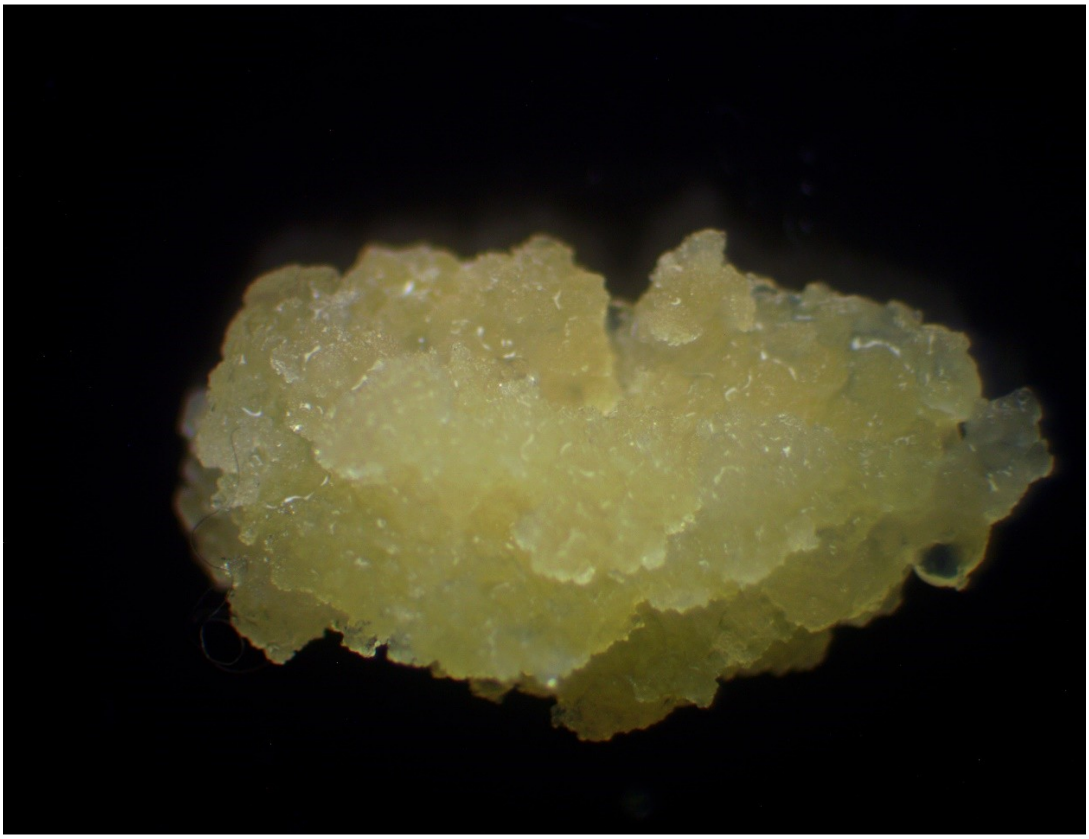

Figure S2. Callus used in this study.

Table S4. Primers used for RT-qPCR.

Primer name	Primer sequence	Primer name	Primer sequence
SaWRKY1F	5′-TGAGAGCAGCAACAACGATAG-3′	SaWRKY1R	5′-CGTTCTTCCCTTGGCTTCTT-3′
SaWRKY2F	5′-GCCTCATCCGCTCATCATAATA-3′	SaWRKY2R	5′-CTCCACCTATAGCCATCATCAAG-3′
SaWRKY3F	5′-CCGCCAAAGAGAGATCACAA-3′	SaWRKY3R	5′-CATTCTCCGTGCCGCTATTA-3′
SaWRKY4F	5′-GAAAGGGAACAAGCCGAAGA-3′	SaWRKY4R	5′-ACCTGTATCCATCCTCCAGAT-3′
SaWRKY5F	5′-TAGAGAGCAAAGGAGGTCAGA-3′	SaWRKY5R	5′-GGGAAGGTGCACTTGTAGTAA-3′
SaWRKY6F	5′-ACGAAGAGACGGACGTATGA-3′	SaWRKY6R	5′-CAACGCTCAACAGAGGAGAA-3′
SaWRKY7F	5′-TCATGAATCGGGTCCCTTTG-3′	SaWRKY7R	5′-TCCAGCGAGAAATCCAATCC-3′
SaWRKY8F	5′-GAGAAGGAACGGCTGATGTTAT-3′	SaWRKY8R	5′-GCTCAGGGCTCTTACTTTGTT-3′
SaWRKY9F	5′-CCCACCTTATCTTCAACGAAGT-3′	SaWRKY9R	5′-AGCTGAGGAGGATGGAGTTA-3′
SaWRKY10F	5′-GAGGAGGAAGAACTGGAATCAG-3′	SaWRKY10R	5′-GAGCTTGGAGAGATCGGTAATC-3′
SaWRKY11F	5′-GCTCAGTCATGTAACCGACAA-3′	SaWRKY11R	5′-CCAAGGCGAAGATCCTCAAA-3′
SaWRKY12F	5′-CTAGTGCTGGATGCTCTGTTAG-3′	SaWRKY12R	5′-ATTGTGCTTTCCCTCGTATGT-3′
SaWRKY13F	5′-AGAAGTGCACCGTGAAGAAA-3′	SaWRKY13R	ATGGACAGTGGTGGTTGTG-3′
SaWRKY14F	5′-ACCTACGCCTTTGACCATAAC-3′	SaWRKY14R	5′-CGGCGATGGACGATGATAC-3′
SaWRKY15F	5′-GTCCGTGTTATCGTCCTCTTC-3′	SaWRKY15R	5′-CTTGGCTTGCTCACTTTGTTAG-3′
SaWRKY16F	5′-ATTCATCGGGCCGTTTCTC-3′	SaWRKY16R	CTTCTTCTTCTCCGAGGGTTTC-3′
SaWRKY17F	5′-ACCCGAGACGATAAGAAGGA-3′	SaWRKY17R	5′-ATTGGTCGCTACTGGTATTGG-3′
SaWRKY18F	5′-GGTTATGATGGCGAAGGAGATG-3′	SaWRKY18R	5′-ACTACTCTGGGTTCTCGTATGG-3′
SaWRKY20F	5′-AGAGGAAGAGGAAGAGGAAGAG-3′	SaWRKY20R	5′-CCGCCGTTAGGGTTTGTAAT-3′
SaWRKY21F	5′-GGGTCGGTTTACGATGAACT-3′	SaWRKY21R	5′-GGAGGAGGAAGGCATGTTATT-3′
SaWRKY22F	5′-TGGGCAGAAGCCAATCAA-3′	SaWRKY22R	5′-GGTCTTCCAAGCATCTCTCTAC-3′
SaWRKY23F	5′-ATCCACCACTTGCTCCAATC-3′	SaWRKY23R	5′-CGGGCACTTCTACTCTTGTTAG-3′

SaWRKY24F	5′-TCCACCCTCAGCACTTAAAC-3′	SaWRKY24R	5′-CTCTTCTCTTACCTCACCCATTT-3′
SaWRKY25F	5′-GCCGGGTCAATATCACTCAA-3′	SaWRKY25R	5′-TCACCACTTTCTGCCCATAC-3′
SaWRKY26F	5′-CCCTGATGATGACAGCCTAAT-3′	SaWRKY26R	5′-GCTCCATTTGCTCTTCCTTTC-3′
SaWRKY27F	5′-GGCTACCGATGGCGTAAATA-3′	SaWRKY27R	5′-ATGGATCTTCTGAGGACCTTTC-3′
SaWRKY28F	5′-CAAGTTCCCAAGAGGCTACTAC-3′	SaWRKY28R	5′-CCGATAAGTGACCTCGAAGATG-3′
SaWRKY29F	5′-ACGTGAAGAAGAGAGTGGAGAG-3′	SaWRKY29R	5′-ATGGCAACTGGTTGAGGTAAAG-3′
SaWRKY30F	5′-AAGCTGAGACAGTGAACGATG-3′	SaWRKY30R	5′-TCCCGCATTTGAACACCTATAA-3′
SaWRKY31F	5′-AGAGGCGGAGAGAGTAGTTATAG-3′	SaWRKY31R	5′-GTTGGAGGGTGATGCAGTAA-3′
SaWRKY32F	5′-GAGGCGAGAGTAGCGTTTATG-3′	SaWRKY32R	5′-GCAGTGGTGCAACGATAGTA-3′
SaWRKY33F	5′-TTCTTCGCCCACTTGTTCTAC-3′	SaWRKY33R	5′-GGAGCCCATAATCGCTCAAA-3′
SaWRKY34F	5′-CCAAATCCAAATGCCTCCAATAG-3′	SaWRKY34R	5′-GGTAATCCTGAACCAGACTGAAA-3′
SaWRKY35F	5′-CCATGTCCACGAGCCTATTATC-3′	SaWRKY35R	5′-GGTGGTTATGTGTGCCTTCA-3′
SaWRKY36F	5′-GCGGAAGCAAGACTCTCATAA-3′	SaWRKY36R	5′-CAGCGGTATCCATCATTCACTAT-3′
SaWRKY37F	5′-CTTCGGAGGAGCTTTCTGATT-3′	SaWRKY37R	5′-CACGGTGGATAGAGCATTCTT-3′
SaWRKY38F	5′-AGTGAGGAAGTGGCTGATAATG-3′	SaWRKY38R	5′-GCTGGCTCTGAAACCCTATT-3′
SaWRKY39F	5′-AAGGTTGAGCGTTCTCTTGAT-3′	SaWRKY39R	5′-CTACCATCTCTTGTTGGCTTACT-3′
SaWRKY40F	5′-GGTGCGAGTTGGATGTGAATA-3′	SaWRKY40R	5′-CTCGTTTCCCTCGTTTCTCTTC-3′
SaWRKY41F	5′-GCTGTGCTCACGACTTATGA-3′	SaWRKY41R	5′-TGGCGGTCTGTTTAGTGTATG-3′
SaWRKY42F	5′-GTCATGGGAGAGTCAGAAAGATG-3′	SaWRKY42R	5′-GGAACCGCAAGGGATTAGTT-3′
SaWRKY44F	5′-GTGGAACGCTCTTATGATGGA-3′	SaWRKY44R	5′-GGCGTCTAGGTTGAGGTTTAG-3′
Fbp1F	5′-TGGCGTGTCCTGTTTCTATC-3′	Fbp1R	5′-CGCACTCCATAGGTTTCTTCT-3′
Fbp3F	5′-CCTCGTGTACTGGGAAATGG-3′	Fbp3R	5′-GCAAGAACGCAATGCCTAAA-3′
ODDF	5′-TTTAGCATTGGGTGGGACTC-3′	ODDR	5′-CTTGGCGATTTGCATTGGTTA-3′
CSAF	5′-GCCAATATACCGAGGACAGAAG-3′	CSAR	5′-CAACCGCAAGATCACAAACAG-3′
FAB1AF	5′-AGCAGTTCTCAAAGGAGCTAAA-3′	FAB1AR	5′-ACCTTCGTGCGACAACTAAA-3′

PP2CF	5′-ACTGACCAGGCAATCCTTTC-3′	PP2CR	5′-ATCCATAACCTTCGGCCATTTA-3′
Atactin2F	5′- CAGAAGGATGCATATGTTGGTG-3′	Atactin2R	5′- TGTAGAAAGTGTGATGCCAGAT-3′

Method S1.

Total RNA was extracted from the leaves of 7-year-old *S. album* trees using Eastep® Super Total RNA Extraction kit (Promega, Shanghai, China) according to the manufacturer's instructions. One μg of total RNA was used to synthesize first strand cDNAs for 3′ or 5′ end amplification with the SMARTer RACE cDNA Amplification Kit (Clontech Laboratories Inc., CA, USA) in 20 μL volume. The two specific primers were designed for completing the *SaWRKY1* 3′ end (F1: 5′-ACCCTACTCAGCCCGA GCAGAA-3′; F2: 5′-GGTGGAACAGATGGCTACC TCA-3′) and 5′ end (F1: 5′- GTTTCTTGTTTTCGGCACTCAC-3′; F2: 5′-CGTGG CGGAGAGGGTTCG-3′) with nested PCR. PCR products were purified using the Gel Extraction Kit (Dongsheng Biotech, Guangzhou, China). Purified fragments were then cloned into pMD-18-T vector (Takara, Dalian, China) and sequenced at the Beijing Genomics Institute (BGI, Shenzhen, China). After sequence comparison, the full open-reading frame of *SaWRKY1* was amplified using gene-specific primers (F: 5′-ATGGATTAC TCTTCTTGGACGGC-3′; R: 5′-TCAA AATTTTTCATTTTGACTTT-3′) by RT-PCR.

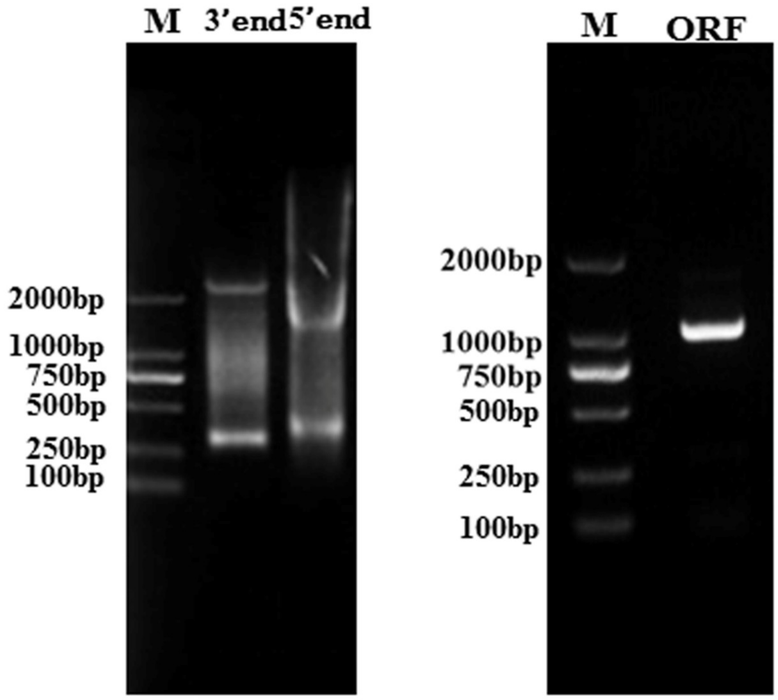

Figure S3. Amplification of 3′ and 5′ ends, as well as the ORF, of *SaWRKY1*.

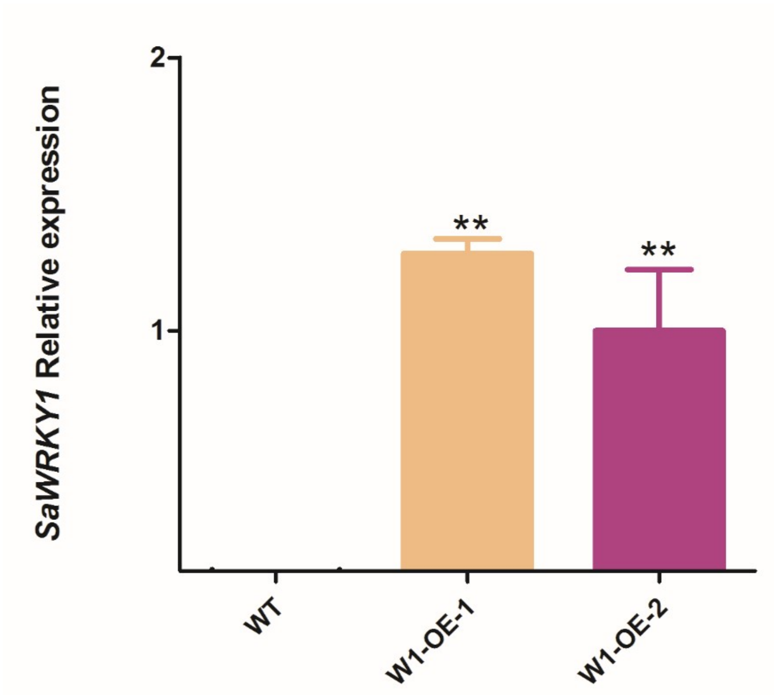

Figure S4. RT-qPCR analysis of *SaWRKY1* gene expression in wild type (WT) and transgenic *Arabidopsis*. Values shown are the means of three independent biological replicates ± SE. Asterisk indicates significance at $P<0.01$ between WT and transgenic lines using Duncan's multiple range test. *Atactin2* (AT3G18780) was used as the internal reference gene.
